# Machine learning based local recurrence prediction in colorectal cancer using polarized light imaging

**DOI:** 10.1117/1.JBO.29.5.052915

**Published:** 2023-11-23

**Authors:** Anamitra Majumdar, Jigar Lad, Kseniia Tumanova, Stefano Serra, Fayez Quereshy, Mohammadali Khorasani, Alex Vitkin

**Affiliations:** a University of Toronto, Department of Medical Biophysics, Toronto, Ontario, Canada; b McMaster University, Department of Physics and Astronomy, Hamilton, Ontario, Canada; c University of Toronto, Department of Laboratory Medicine and Pathobiology, Toronto, Ontario, Canada; d University of British Columbia, Department of Surgery, Victoria, British Columbia, Canada; e University of Toronto, Department of Radiation Oncology, Toronto, Ontario, Canada

**Keywords:** polarimetry, Mueller matrix microscopy, artificial intelligence, supervised machine learning, cancer prognosis, outcome prediction

## Abstract

**Significance:**

Current treatment for stage III colorectal cancer (CRC) patients involves surgery that may not be sufficient in many cases, requiring additional adjuvant systemic therapy. Identification of this latter cohort that is likely to recur following surgery is key to better personalized therapy selection, but there is a lack of proper quantitative assessment tools for potential clinical adoption.

**Aim:**

The purpose of this study is to employ Mueller matrix (MM) polarized light microscopy in combination with supervised machine learning (ML) to quantitatively analyze the prognostic value of peri-tumoral collagen in CRC in relation to 5-year local recurrence (LR).

**Approach:**

A simple MM microscope setup was used to image surgical resection samples acquired from stage III CRC patients. Various potential biomarkers of LR were derived from MM elements via decomposition and transformation operations. These were used as features by different supervised ML models to distinguish samples from patients that locally recurred 5 years later from those that did not.

**Results:**

Using the top five most prognostic polarimetric biomarkers ranked by their relevant feature importances, the best-performing XGBoost model achieved a patient-level accuracy of 86%. When the patient pool was further stratified, 96% accuracy was achieved within a tumor-stage-III sub-cohort.

**Conclusions:**

ML-aided polarimetric analysis of collagenous stroma may provide prognostic value toward improving the clinical management of CRC patients.

## 1 Introduction

Colorectal cancer (CRC) remains both the third leading cause of cancer-related deaths and the third most commonly diagnosed cancer globally.^1^^,^^2^ With an estimated 1 million cases per year,^3^ the associated burden is expected to increase by 60%, approaching 2.2 million new cases and 1.1 million annual deaths by 2030.^2^ The primary curative treatment for localized tumors is surgical resection.^3^ However, it is shown to be less effective in patients with more advanced disease, where adjuvant therapy is shown to improve survival.^1^^–^^3^ In more advanced tumor-node-metastasis (TNM) stages (e.g., TNM stage III), recurrence and more specifically, local recurrence (LR) plays an important role in determining unfavorable patient outcomes.^4^ The ability to identify stage III CRC patients at risk of LR following standard therapy presents an opportunity for creating more personalized care and helping to avoid the over- or under-treatment of patients.

To accomplish this, prognostic biomarker curation has largely focused on molecular and genetic indicators.^5^^–^^9^ In recent years, various commercial testing kits have emerged for predicting risk of distant recurrence in stage II and III CRC patients (e.g., OncotypeDx, ColoPrint, ColoGuideEx, and ColoGuidePro). However, their suboptimal accuracy and/or high costs continue to drive the search for alternative prognostic markers.^8^^,^^9^ For example, there is increased evidence suggesting valuable prognostic information exists within the growth patterns of peri-tumoral collagen, a crucial component of the tumor microenvironment (TME).^10^^–^^13^ Known as the desmoplastic response (DR), this growth and structural remodeling of connective tissue has been shown to correlate with 5-year relapse-free survival and LR.^14^^–^^17^ DR utilizes a three-class categorization of stromal maturity (immature, intermediate, and mature). However, given the qualitative and subjective nature of its assessment, DR has not witnessed widespread clinical adoption primarily due to inter-observer variability.

Optical techniques allow for quantification of DR and collagen assessment via various modalities for applications in fields, such as oncology, cardiology, and dentistry.^18^ The current gold standard, second-harmonic generation, is specific to collagen, but its high cost, lengthy imaging times, modest fields of view, and overall complexity restrict its use to research applications.^19^^,^^20^ Analogous considerations limit techniques, such as scanning electron microscopy and optical coherence tomography (excluding ophthalmology).^18^^–^^21^ More practical, staining techniques such as Mason’s trichrome and picrosirius red that preferentially bind to collagen can easily be implemented with current pathology microscopes.^22^ However, concerns about additional staining, expense, (in)compatibility with current histology workflows, reproducibility, quantification, and information content of scoring systems prevent these staining approaches from being regular additions to histology departments.^22^^,^^23^ Alternatively, polarized light microscopy (PLM) offers a simpler approach with an ability to obtain high contrast images suitable for quantification from unstained tissue samples.^24^ PLM addresses many of the above-mentioned concerns, and consequently has been applied in breast, cervical, prostate, brain, and colon cancers.^25^ More specifically, a PLM technique known as Mueller matrix (MM) polarimetry has been increasingly combined with machine learning (ML) algorithms to directly correlate the underlying biological phenomena with their polarization properties to identify prognostically relevant parameters.^26^^–^^31^

Our recent studies utilized rotating crossed linear polarization microscopy,^32^^–^^36^ combined with an unsupervised ML clustering pipeline,^32^ the latter demonstrating appreciable correlations with 5-year patient survival outcome.^32^ However, in that study no associations were investigated with another important clinical endpoint (5-year LR), and the unsupervised learning algorithm used was not easily scalable. Our more recent study using full MM polarimetry demonstrated the presence of statistically significant correlations between certain MM parameters and 5-year LR status.^37^ However, no predictive analyses were made to further investigate the prognostic value of the MM polarimetric approach. Building on these prior works, the present study thus aims to assess whether MM parameters hold any prognostic value in relation to 5-year LR status by integrating supervised ML to enable robust feature extraction and outcomes prediction on the same acquired dataset. In this paper, we thus describe the (1) acquisition and extraction of various MM parameters of potential interest; (2) process of identifying the most relevant ones as determined by the supervised learning algorithm; and (3) conduct performance analyses (sensitivity, specificity, total accuracy, and area under receiver operating characteristic curve) to assess the overall performance of this refined approach. The findings suggest the prognostic value of MM polarimetric parameters in combination with supervised learning as a low-cost, quantitative, and stromal-based predictive tool for LR stratification in stage III CRC patients.

## 2 Methods

### 2.1 Ethics

University Health Network (Toronto, Ontario, Canada) provided ethics approval. Given the retrospective nature of the study along with patient anonymization, patient consent was waived by the ethics board. All procedures and handling of patient data were conducted in accordance with the University Health Network Research Ethics Board guidelines/approvals.

### 2.2 Patient Tissue Samples

This study employed a total of 38 archival surgical resection samples acquired from stage III left-sided CRC (sigmoid-rectal) patients prior to receiving adjuvant chemotherapy. Relevant patient clinical outcome data (i.e., 5-year LR status) were used to assess correlations. For 5-year LR-status, 29 patients exhibited no-LR whereas 9 patients showed signs of LR at this time-point. Each patient sample comprised a pair of 4.5  μm thick unstained sections extracted from formalin-fixed and paraffin-embedded tissue blocks. For polarimetric analysis, sample preparation involved chemical dewaxing to avoid possible polarization artifacts.^32^^–^^37^ No further processing was required for polarimetric imaging. Adjacent slides were hematoxylin and eosin (H&E) stained and imaged at 20× magnification on an Aperio ScanScope CS (Leica Biosystems, United States) for the pathologists’ region-of-interest (ROI) selection.

### 2.3 ROI Selection and Histology

**Fig. 1:**
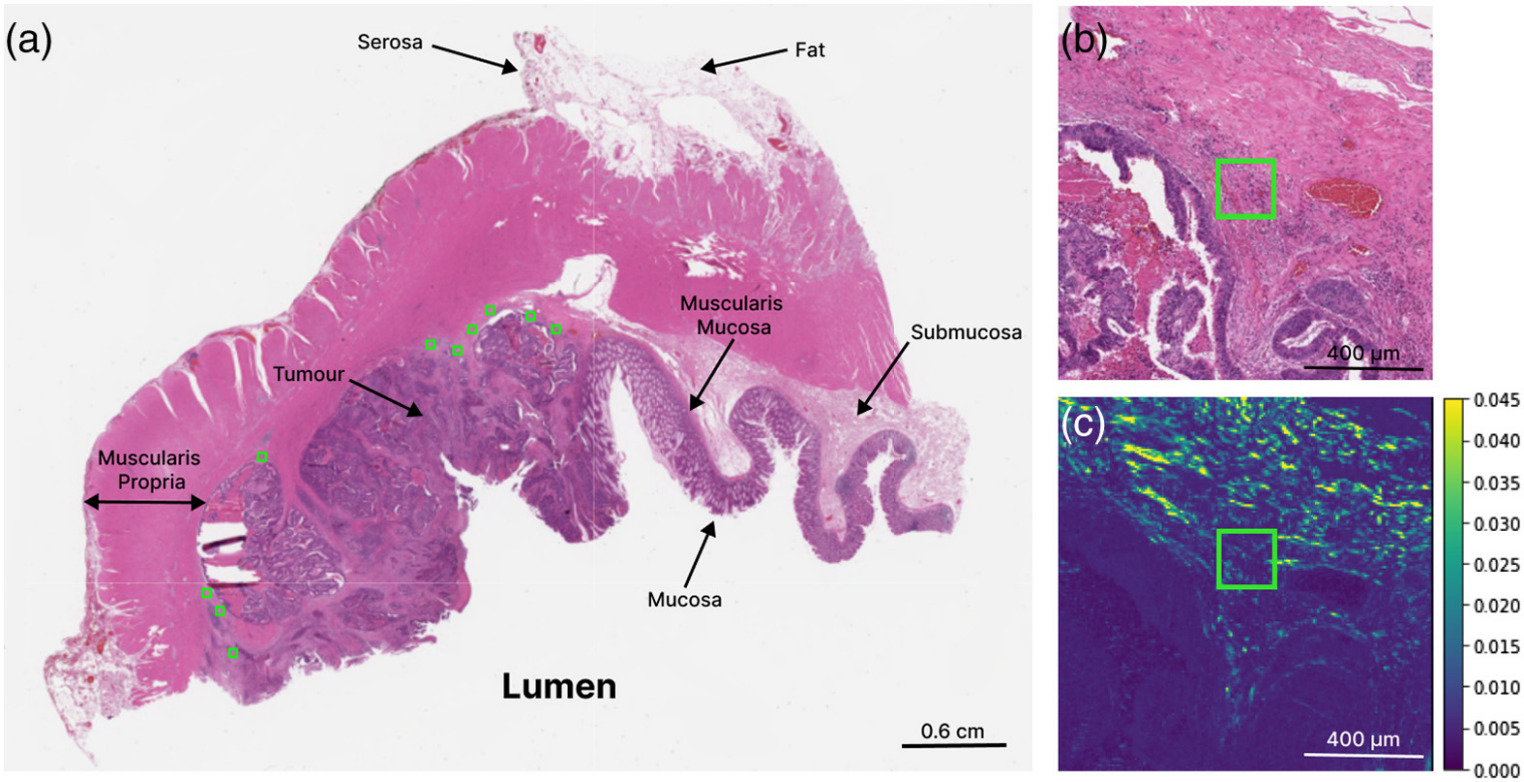
Whole-slide and ROI-based histologic and polarimetric imaging of a stage III CRC sample for qualitative ROI-based analysis with respective tissue labels. (a) H&E-stained slide acquired at 20× magnification (b) H&E and (c) zoomed-in images at 80× magnification of MM element M43 showing the region around the right-most ROI in panel (a). The brighter areas in panel (c) represent birefringent tissues that contain more collagenous stroma. Green squares represent pathologist-identified ROIs chosen along the invasive tumor front.

A total of 356 ROIs (∼200  μm×200  μm) were selected on the 38 adjacent H&E-stained samples by an experienced gastrointestinal pathologist. To avoid selection bias, the pathologist was blinded to the polarimetry images and clinical outcome data. Along the tumor invasive front, number per patient slide ranged from 3 to 14 ROIs depending on tumor size and stroma morphology. Furthermore, ROI selection at this interface was made to comprise of relevant collagen structures while excluding other components (e.g., cancer cells and surrounding smooth muscle tissue) as they are not part of the analysis (Fig. 1). ROI size was determined by balancing the need for robust statistics, capturing stromal spatial heterogeneity, and maintaining adequate spatial resolution.^33^^,^^34^ Visual tissue landmarks were used to guide imaging and transfer these ROIs from the H&E images to the polarimetric images for analysis. Image processing and analysis were then performed using Python.

### 2.4 Polarimetric Image Acquisition

**Fig. 2:**
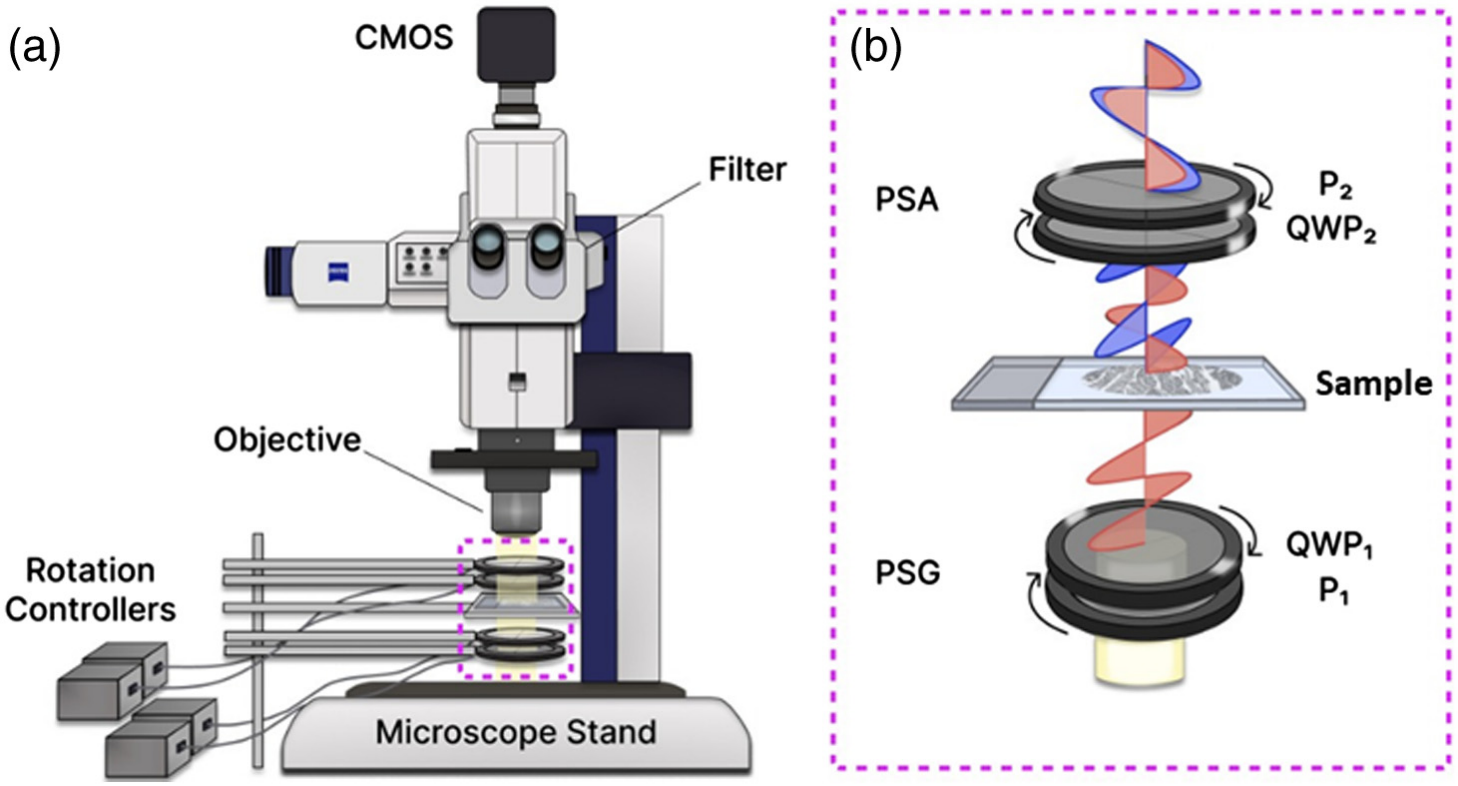
(a) Schematic of the widefield inverted microscope and (b) MM polarization optics. Polarization state generation and analysis modules (PSG and PSA, respectively) are both comprised of a counter-rotating polarizer (P) and QWP pair. Upon interacting with birefringent components of tissue (e.g., collagen fibers), the polarization state of transmitted light is altered [illustrated diagrammatically in panel (b)] and detected via the PSA, providing a source of useful tissue contrast.

Imaging was done using a multiscale Mueller polarimetry methodology previously developed by our group (Fig. 2).^38^ It involves incorporating a pair of linear polarizers (LPVISE100-A, Thorlabs) and quarter-wave plates (QWPs) (AQWP05M-600, Thorlabs) on computer controlled motorized rotation mounts (PRM1.MZ8, Thorlabs) into the beam path of an AxioZoom V16 stereomicroscope (Zeiss, Germany) [Fig. 2(a)]. These polarizer-QWP pairings rotate in both directions and are known as polarization state generator (PSG, incident beam) and polarization state analyzer (PSA, transmitted beam) [Fig. 2(b)]. Illumination is achieved with 310 W uncollimated white light source [Illuminator HXP 200C (D), Zeiss] passing through a collimator and a 630 nm filter (ET630/75 or ZET630/10, Chroma); the filter helps reduce unwanted scattering and hemoglobin absorption.^39^ Signal is captured by a digital CMOS camera (ORCA-Flash4.0 V3, Hamamatsu) with 2048×2048  pixel array. Images were obtained at 80× magnification with a field-of-view/lateral resolution of $1.66\times 1.66\;{\mathrm{mm}}^{2}/2.2\;\mu m$.

To capture all available polarization information and improve signal-to-noise, a total of 24 images per sample at specific angular orientations of PSG and PSA were acquired to directly calculate the Stokes vectors and MM elements (discussed briefly in subsequent section).^38^ The MM elements were calculated for each individual pixel within a given ROI (∼60,000) to capture more detailed information of the peri-tumoral collagen fiber; note that the system’s optical resolution is within the typical thickness range of these fibers (1 to 20  μm).^40^ Calibration was performed by calculating MMs for air and retarders to account for artifacts that may arise due to off-axis light reaching the objective as result of the widefield beam.^38^ Slight image shifts and loss of focus may also occur as the polarizers and waveplates rotate to generate the various polarization states. The Python library pyStackReg, which performs image co-registration, was used to account for such minor artifacts.^41^

### 2.5 Polarimetric Feature Extraction

**Fig. 3:**
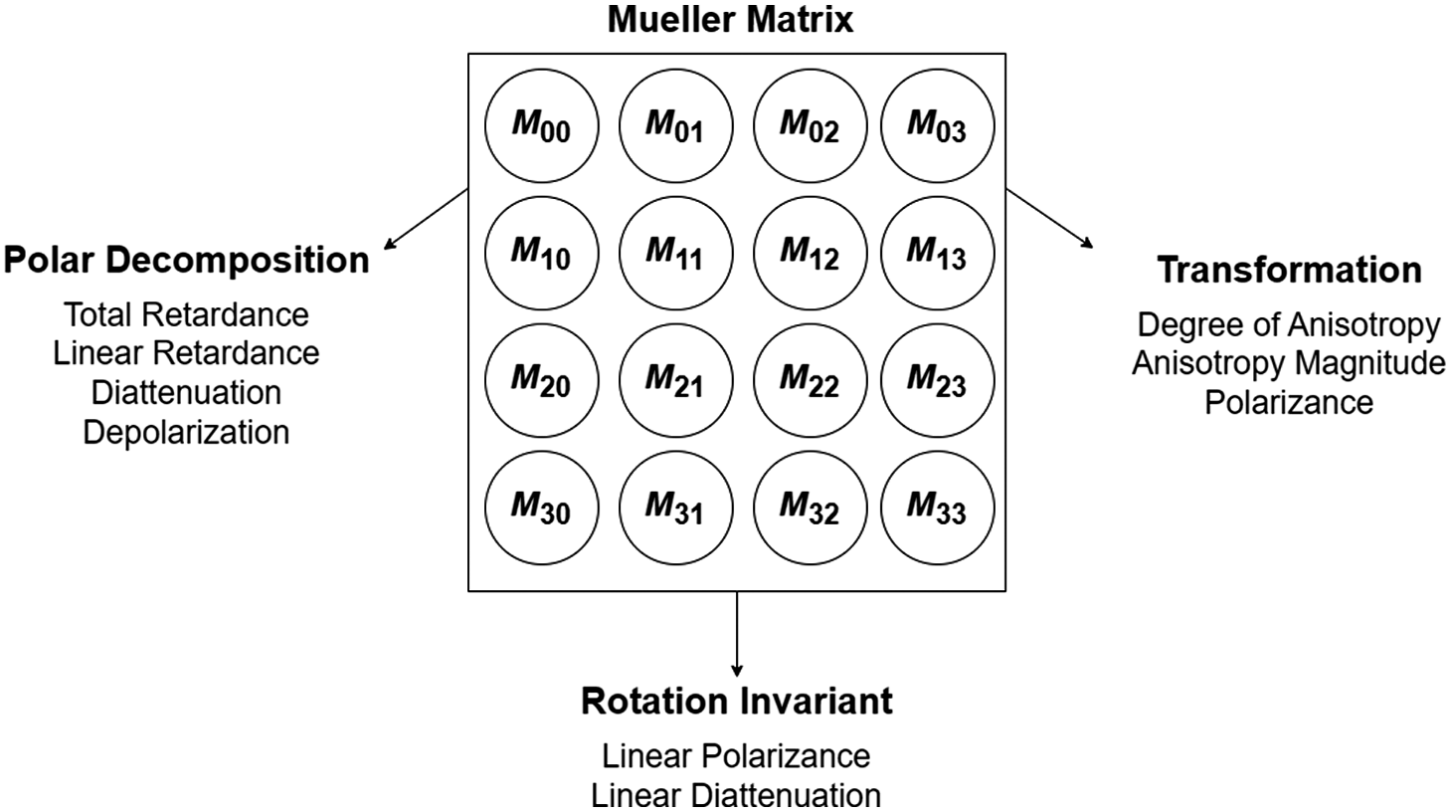
The 25 features extracted from polarimetric images for analysis. These arise from four primary categories based on their origin: original 16 MM elements, Lu–Chipman MM polar decomposition, MM transformation, and MM rotation invariant properties.

Unlike the prior rotating-crossed-linear-polarizers methodology,^32^^–^^36^ which yield a limited (but nevertheless telling) set of polarization signals, the MM approach yields more polarization information and may provide biophysical insight into the polarized light-tissue interactions based on the Stokes–Mueller formalism. As the name suggests, full MM analysis requires both Stokes vectors and the MM transfer function to express properties of the polarized light states upon interaction with the medium.^24^ This is described by a linear relationship

$$S_{o}=MS_{i},$$ (1)

$$[\begin{array}{c} I_{o}\\ Q_{o}\\ U_{o}\\ V_{o} \end{array}]=[\begin{array}{cccc} M_{00} & M_{01} & M_{02} & M_{03}\\ M_{10} & M_{11} & M_{12} & M_{13}\\ M_{20} & M_{21} & M_{22} & M_{23}\\ M_{30} & M_{31} & M_{32} & M_{33} \end{array}][\begin{array}{c} I_{i}\\ Q_{i}\\ U_{i}\\ V_{i} \end{array}],$$ (2)

where $S_{i}$ and $S_{o}$ is the Stokes vectors of the incoming and the outgoing polarized light [Eq. (1)]. In Eq. (2), I is the total detected light intensity, Q is the difference between the horizontal and vertical linear polarization states, U is the difference between the 45 deg and 135 deg linear polarization states, and V is the difference between right and left circularly polarized light. M represents the Muller matrix, which contains 16 elements encapsulating tissue polarization properties (for example, depolarization, diattenuation, retardance, etc.). To extract such biophysically relevant properties and others, various linear algebra techniques are required. Promising approaches based on their relative simplicity and biophysical value^24^ include Lu–Chipman polar decomposition,^42^ MM transformation,^43^ and MM rotation transformation (rotation invariant properties).^44^ With their 9 resultant features plus the 16 MM elements summarized in Fig. 3, the dataset contains a total of 25 features across the 356 acquired ROIs from 38 different patient samples.

Important to note that a major consequence of this ROI-sample imbalance is the existence of a single binary outcome label (i.e., LR versus non-LR patient). Consequently, this necessitates aggregating all data to a single patient-level output. Hence, there are two data-aggregation checkpoints along the path to making the polarimetric data and sample labels equivalent, with the first being discussed here and the second in the following section. The first checkpoint is at the pixel-ROI level. With ∼60,000 MM results acquired in a single ROI, this presents the opportunity for robust and simple summary statistics in the form of median values. For each ROI, a median MM is produced comprised of 16 median-MM elements, which then undergo the various feature extraction methods. The advantage of the median over alternative methods (e.g., distribution-asymmetry analysis) is its simplicity in interpretability and resistance to influence from outliers,^45^ which is key to developing sound ML models; however, the possible loss of detailed information inherent in this averaging must also be borne in mind.

### 2.6 Machine Learning Methods

Prior published polarimetric findings utilized unsupervised clustering methods to assess overall survival.^32^ Although appropriate given the sample size constraints, it is a platform that is not easily scalable to larger and more complex multivariate datasets. The natural progression with increasing sample numbers is toward supervised ML methods as these can perform predictive analyses, elucidate the most valuable features, and easily scale to operate on larger datasets. Supervised learning (and ML overall) forms two primary groups: classifiers and regressors. The former involves problems that are discrete or categorical in nature, whereas the latter deals with continuous (e.g., time-series) datasets.

The current problem requires a classifier, given its categorical nature (i.e., LR versus no-LR). There exist several suitable algorithmic approaches; in this study, we considered logistic regression, linear discriminant analysis (LDA), support vector machine (SVM), random forest, and extreme gradient boosting (XGBoost), each with its unique advantages and drawbacks. Briefly, logistic regression maps the probability of a binary outcome (i.e., 0 or 1) in the form of logarithmic odds.^46^ LDA separates two or more classes by determining the linear combination of features that best achieve the task.^46^ SVM attempts to linearly separate data into a two-class problem via hyperplanes, finding the most optimal margin (or distance) from the plane to the nearest data point from either side.^46^ Random forest is an amalgamation of multiple decision trees [essentially a flow chart where one attempts to find the optimal nodes that represent the target leaf nodes (outcomes)] where the mode serves as its output.^46^ XGBoost is an optimized library of gradient boosted decision tree algorithms – an ensemble technique whereby new decision trees are sequentially introduced to minimize error (gradient descent) and make the final prediction.^47^ Compared to the alternatives, XGBoost offers many advantages including better handling of (1) class imbalances and overlapping classes; (2) presence of outliers; (3) correlated inputs; and (4) directly extracting relative feature weights. In fact, it is consistently one of the most employed and successful algorithms during competitions.^47^ However, it is not without some key caveats, notably that XGBoost is difficult to interpret given its complexity and its performance is highly dependent on the tuning of hyperparameters (parameters that influence model performance but are external to the model and data). If not done correctly, this risks overfitting XGBoost to the dataset.^48^

To enhance the effectiveness and performance of the ML model, outlier assessment was implemented. This involved iteratively training the model with all possible subsets of the samples and eliminating ones that negatively affect model performance. This approach allowed the algorithms to focus on the more representative patterns and relationships within the dataset, ultimately enhancing its predictive capabilities (XGBoost results before vs. after elimination of outliers: total accuracy 73% versus 78%, area under the receiver operating characteristic curve (AUROC) 72% versus 77%, sensitivity 31% versus 50%). Consequently, three samples were removed from the study cohort (i.e., leaving 35 out of 38 patients and 333 out of 356 ROIs). Eliminating these outliers reduces the potential to introduce noise and unnecessary bias in the training process.

Given the relatively small(er) dataset after accounting for outliers [333 ROIs from 35 patients—no-LR = 263 ROIs (27 patients); LR = 70 ROIs (8 patients)], a standard 80:20 train-test split ratio [train: ∼267 ROIs (28 patients); test: ∼66 ROIs (7 patients)] was used to provide an adequate learning opportunity for the model. Feature selection through identifying relative feature importance was also undertaken during the training phase to improve performance. Reported results are in the form of averages calculated over five different training-test set combinations (fivefold cross-validation) to avoid selection bias and test model performance on all parts of the dataset. There is no intra-ROI contamination between the training and testing sets (i.e., within-same-patient ROIs are kept together). This is all conducted at the ROI-level where the recurrence status is assigned to each region, and it is treated as an individual data point. The second checkpoint, at the ROI-patient level, is now introduced upon reaching the final patient-level evaluation in the form of a simple majority-vote scheme. In this instance, a 50% threshold is applied: if 50% or more of the ROIs for a patient in the test set are predicted as one class (i.e., locally recurring or not locally recurring), then the patient is considered to belong to that class by majority-vote. This classification then serves as the model’s predicted outcome and is compared to the ground truth patient labels in the test set for analysis.

### 2.7 Performance Assessment

As a result of the binary categorical nature of the LR-outcome data, a 2×2 confusion matrix (true outcome versus predicted outcome) was formed to determine model performance for each cross-validation run. From this, the means of total accuracy, sensitivity, and specificity were calculated given the true/false positive/negative rates in each matrix. The culmination of all cross-validation results is then summarized into an ROC curve where the mean AUROC is used to characterize overall model output.

## 3 Results and Discussion

This study improves upon previous efforts^32^^–^^37^ to create a polarimetric ML workflow adept at providing valuable prognostic insights, by (1) utilizing a wider set of more robust optical polarimetric features and (2) using scalable supervised learning techniques. Specifically, the recent publication of Tumanova et al.^37^ used Matt–Whitney U-test inferential statistics to demonstrate that certain MM parameters do offer some separation between LR and no-LR groups; however, no predictive analyses were performed. Here, implementing supervised learning allows polarimetric features to be selected and assessed for their prognostic value, yielding differences in methodology, analysis, and final results. Ultimately, different conclusions are drawn with respect to LR-relevant MM parameters, and some overlap between the two studies point to suggest interesting insights as briefly highlighted later in this section.

**Fig. 4:**
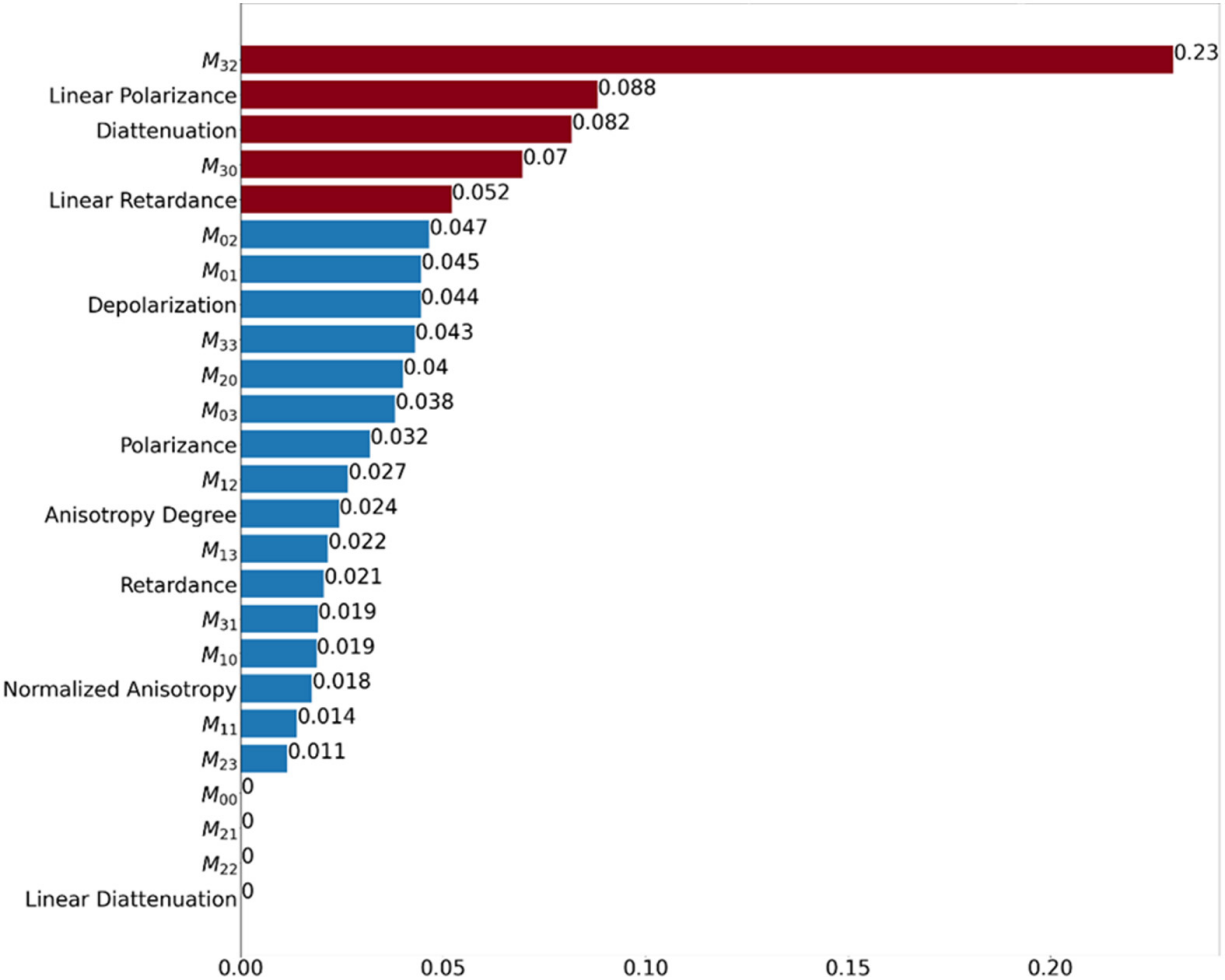
Relative feature importance plot for XGBoost as determined during the training phase. MM features are listed on the y-axis with respective weightings on the x-axis achieved using minimum-maximum normalization. Red bars indicate the top five most relevant features according to their weights.

Preliminary efforts involved incorporating all 25 MM features as model inputs, which provided only adequate results (Table 1). At the ROI-level for XGBoost, sensitivity and specificity were 50%±24% and 85.6%±4%, respectively, with a total accuracy of 78.1%±4% and a mean AUROC of 77%±13%. When applying the majority-vote scheme to assess performance at the patient level, these metrics essentially remained the same (e.g., total accuracy of 77.9%±11%). The remaining models all demonstrated higher specificities but with significantly lower sensitivities; for instance, logistic regression, which failed to correctly identify a single true LR patient. At this stage, random forest presented the best mean AUROC (80%±11%) but only slightly better than XGBoost, which had a 20% higher sensitivity. In Fig. 6(a), this difference in sensitivities between each model is more clearly depicted; it is evident that this is the distinguishing factor as the remaining performance metrics are rather similar throughout. For our purposes, the preferred model would be balanced in correctly identifying both LR and no-LR patients and here XGBoost appears the marginal “winner”; however, its sensitivity values are modest. It was therefore suspected that not all features were beneficial for the learning algorithm, with some potentially contributing towards noise as opposed to useful unique signal information. One can then utilize feature selection, a process that can optimize ML performance as not all features have equal impact. Indeed, a common practice upon completion of a model’s training phase is the extraction of relative feature importance (typically in the form of coefficients or weights). When applied to this dataset (Fig. 4), the top five most important features across all learning models were (in order of importance): (1) MM element $M_{32}$; (2) linear polarizance; (3) diattenuation; (4) MM element $M_{30}$; and (5) linear retardance.

**Table 1:** Prediction performances of five different ML algorithms examined in this study for 5-year LR (all feature and T-stage combinations). Results are quantified via sensitivity, specificity, total accuracy and AUROC (all averaged over the fivefold cross-validation runs). A total of ∼66 ROIs from seven samples of the test set were analyzed. We used 25 features considering all T-stages (35/35 patients, upper line in each model row), top five features considering all T-stages (35/35 patients, middle line), and top five features for only T-stage 3 (24/35 patients, lower line). Standard deviations indicate their variability/spread across the cross-validation runs. XGBoost (top model row) appears optimal for this classification task; for details, see text.

Model	Parameter space	Sensitivity	Specificity	Total accuracy (ROI)	Total accuracy (patient)	AUROC
XGBoost	All features (all T-stages)	50% ± 24%	86% ± 4%	78% ± 4%	78% ± 11%	77% ± 13%
	Top features (all T-stages)	53% ± 24%	90% ± 6%	82% ± 10%	86% ± 13%	82% ± 10%
	Top features (T-stage 3)	70% ± 0%	99% ± 1%	96% ± 5%	96% ± 7%	99% ± 0%
Random forest	All features (all T-stages)	31% ± 33%	91% ± 5%	79% ± 6%	84% ± 10%	80% ± 11%
	Top features (all T-stages)	43% ± 30%	90% ± 4%	81% ± 7%	86% ± 9%	81% ± 10%
	Top features (T-stage 3)	4% ± 3%	100% ± 0%	90% ± 4%	87% ± 5%	53% ± 1%
SVM	All features (all T-stages)	7% ± 6%	98% ± 4%	79% ± 7%	77% ± 6%	79% ± 15%
	Top features (all T-stages)	10% ± 7%	98% ± 4%	79% ± 6%	77% ± 6%	79% ± 12%
	Top features (T-stage 3)	4% ± 7%	100% ± 0%	90% ± 4%	87% ± 5%	68% ± 21%
LDA	All features (all T-stages)	31% ± 28%	94% ± 6%	81% ± 6%	86% ± 9%	60% ± 20%
	Top features (all T-stages)	31% ± 37%	99% ± 1%	85% ± 8%	86% ± 9%	62% ±19%
	Top features (T-stage 3	17% ± 13%	100% ± 0%	92 ± 2%	92 ± 1%	80 ± 18%
Logistic regression	All features (all T-stages)	0% ± 0%	99% ± 1%	79% ± 8%	77% ± 6%	64% ± 17%
	Top features (all T-stages)	0% ± 0%	100% ± 0%	79% ± 8%	77% ± 6%	37% ± 13%
	Top features (T-stage 3)	4% ± 3%	100% ± 0%	90% ± 4%	87% ± 5%	24% ± 0%

**Fig. 5:**
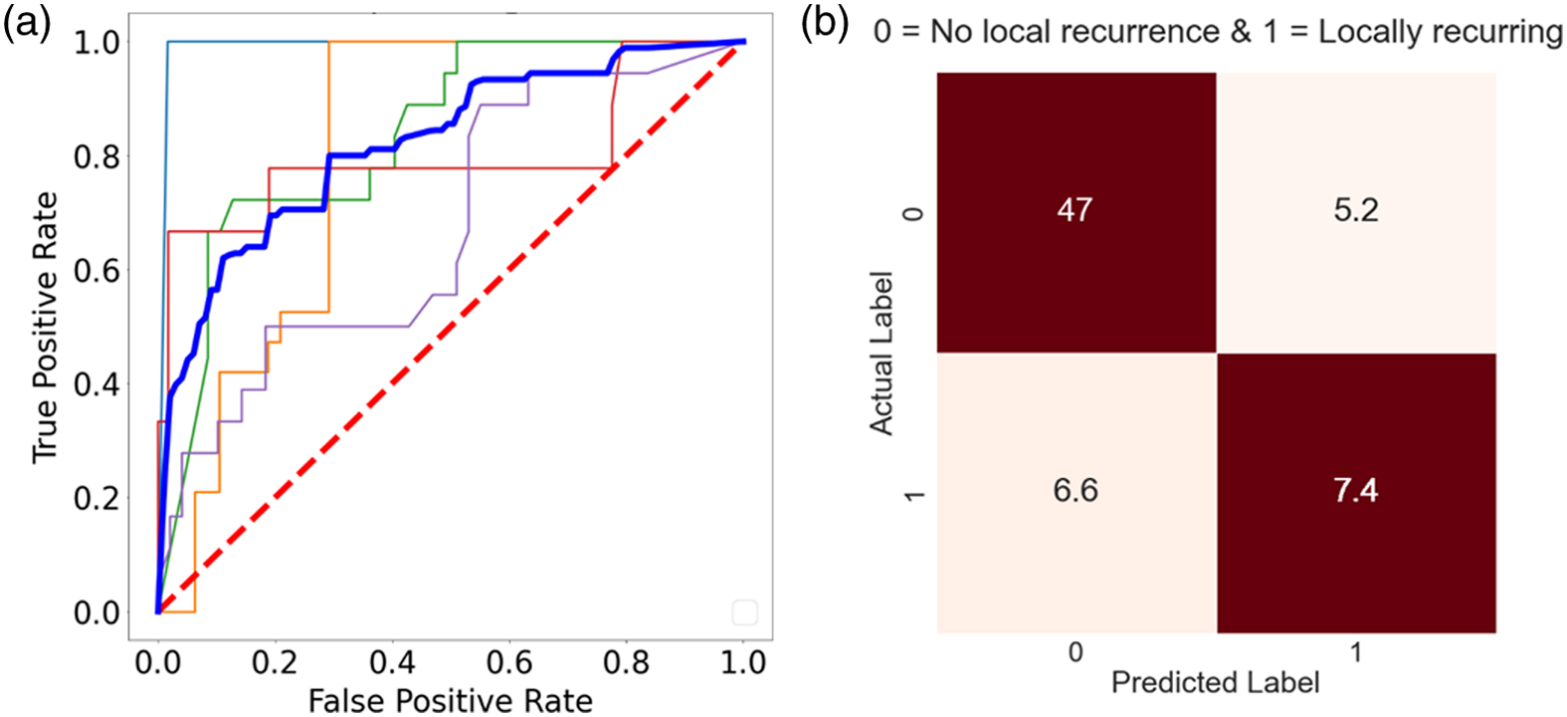
Illustration of XGBoost prediction performance for 5-year LR using the top 5 features: (a) Receiver operating curve. Thick blue line is the mean ROC curve; other thinner lightly shaded lines are the individual ROC curves corresponding to each of the fivefold cross validation runs. The red dashed line represents an AUROC value of 0.5 (∼random chance of predicting the correct class). (b) Confusion matrix. Red squares = correct predictions, white squares = incorrect predictions. Decimal values are due to averaging the five confusion matrices generated from the fivefold cross validation.

**Fig. 6:**
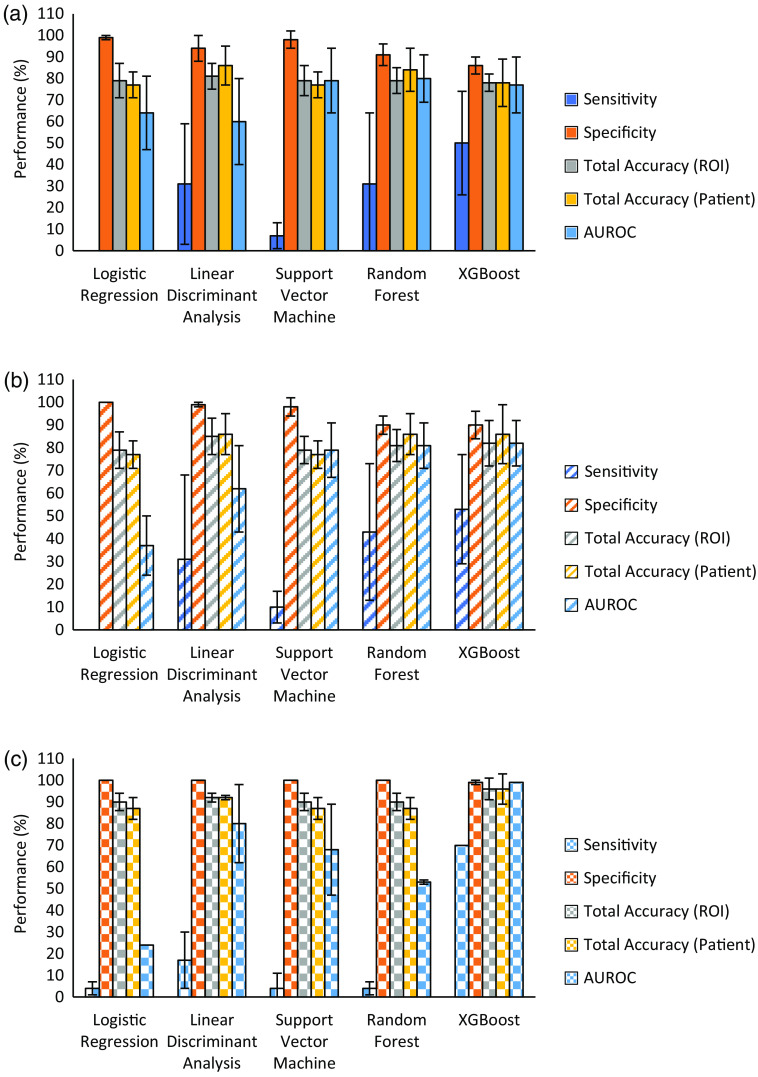
Comparison of all model performances via sensitivity, specificity, total accuracy (ROI), total accuracy (patient) and mean AUROC for when (a) all features and T-stages are assessed, (b) top 5 features and all T-stages are assessed, and (c) top five features in patients with T-stage III are assessed. Error bars are the percent uncertainties for each metric (from variability/spread across the cross-validation runs).

Feeding only these five features into each model does indeed provide a noticeable improvement in results (Table 1). Focusing on XGBoost, as evident from the mean ROC curve in Fig. 5(a), at the ROI-level the model is more capable in distinguishing between the no-LR and LR groups with a mean AUROC of 82%±10%, a 5% improvement compared to when all features are used. This translates to correctly identifying 47 no-LR ROIs (top red square) and 7.4 LR ROIs (bottom red square) on average across the 5-cross validation runs [Fig. 5(b)]. Consequently, there are a higher number of true positives (correctly identified LR ROIs) and true negatives (correctly identified no-LR ROIs) compared to our first attempt utilizing all 25 features, whereby the sensitivity and specificity improve by ∼2% (52.9%±24%) and ∼5% (90.2%±6%), respectively. The total accuracy is 82.3%±10% (∼4% increase). However, unlike the first attempt where the patient-level accuracy was comparable to the ROI-level results, here an almost 10% improvement is observed (i.e., total accuracy of 86.1%±13%). The same cannot be said for the other approaches. Models, such as logistic regression, LDA, and SVM, demonstrated no real benefits from feature importance selection. In fact, contrary to expectation, logistic regression actually experienced a 27% decrease in its mean AUROC performance compared to no-feature-selection approach. It is of note that random forest is the only other model to exhibit some noticeable improvement, for example its sensitivity increasing by 12% (Table 1). From these results, it is evident that XGBoost and random forest are the best candidates for the task at hand. Despite the use of feature importance selection, these improvements in their respective performance metrics are somewhat offset by the relatively large uncertainties, specifically for the sensitivity [Fig. 6(b)], such that the overall performance of these two models is essentially indistinguishable.

Since the current clinical prognostic insights rely in part on TNM staging, it was also of interest to see whether this workflow would benefit any sub-group and thereby also improving model performance, for example as stratified by T-stage. There has been research demonstrating a potential correlation between the local size and spread of the tumor (T-stage) and LR, with patients at higher-risk having more advanced T-stages (3 or 4).^49^^–^^51^ Our N=35 cohort has patients with T-stages ranging from 1 to 4, with 24 assessed having a T-stage of 3 [no-LR = 21 (203 ROIs); LR = 3 (23 ROIs)]. Re-running the models on this redacted N=24 patients data set, performance metrics improved further only for XGBoost (Table 1): specificity=99.0%±1%, sensitivity=70.0%±0%, AUROC=99%±0%, ROI-level accuracy=96.1%±5%, and patient-level accuracy=95.8%±7%. Thus, prediction of LR is the most accurate for this sub-group of T-stage III CRC patients using the developed polarimetric imaging + ML analysis methodology, evidenced by the improved sensitivity and mean AUROC values and reduced uncertainties [Fig. 6(c)]. Unlike these significant XGBoost improvements, the remaining models all performed poorly on their respective metrics (e.g., random forest sensitivity=4.3%±3.1% and AUROC=52.9%±1.4%). The consistent solid performance of XGBoost for all evaluated feature and patient-group combinations demonstrates its robustness and promise for correctly prognosticating which patients will exhibit LR and which will not 5 years into the future. Overall, these results are encouraging, as predicting and mitigating recurrence is of primary concern for effective treatment of stage III CRC patients; with no current commercial toolkit capable of such a task, our technique offers promise towards a low-cost alternative with further development.

With the recent shift in MM research from direct feature engineering and statistics to incorporating more ML prediction,^26^^–^^31^ this study further demonstrates the benefit of ML implementation for gleaning key prognostic MM features toward expanding its application of MM in medicine, particularly for cancer. Prior efforts in this space have largely focused on elucidating certain purportedly biologically relevant MM biomarkers (e.g., polar decomposition and transformation parameters linked with collagen alignment and density^50^ and tumor characteristics^26^^–^^30^^,^^52^^–^^54^). For example, MM features, such as total and linear depolarization, total and linear retardance, diattenuation, etc., have been suggested as relevant for cancer identification.^53^^,^^54^ Although the task of predicting 5-year-distant LR is rather different, it is interesting and reassuring to observe that some of those same features prove useful here as well (e.g., diattenuation and linear retardance, as per Fig. 4). Furthermore, our recent study^37^ also suggested that, for example, diattenuation is a relevant feature for 5-year LR versus no-LR stratification, which is supported by the prognostication results of this analysis. It was thus interesting to note some overlap in the significant polarimetric features revealed by the two approaches but also to note that there were differences. This likely stems from differences in the methodologies and in what the two approaches deliver (correlation versus prediction), and thus which features are most suitable for these two tasks. Overall this line of research demonstrates the value of MM analysis to quantitatively identify and use task-specific (e.g., tumor delineation, 5-year outcome prediction, etc.) biological image features. Nonetheless, more substantive MM imaging + ML analysis studies focusing on treatment outcome prognosis – larger numbers of patients, streamlined and objectivized ROI selection process, improved pixel/ROI /patient level results averaging, optimized ML model prognostication performance, etc.,—will be required to better understand, refine, and potentially deploy this promising technology in the clinic.

The reported findings (Table 1) show promise, but they are not without limitations, chiefly the limited dataset size and class imbalances. With only 35 patient samples assessed and the respective 333 ROIs treated as individual data points, the size is still not well suited for developing truly robust ML models, hence the large uncertainties and overlap. Overfitting may still be of concern with datasets in the low hundreds, despite mitigation efforts through cross-validation. Furthermore, given the prognostic aim of the analysis, it is eventually the patient-level outcome that is of concern, which significantly bottlenecks the workflow by ultimately reducing the number of available data points. There is also the risk of learned bias by having ∼3/4 of the cohort with no LR. Thus, it is unsurprising that the specificity was consistently higher and demonstrated the most improvements across all models and attempts, whereas the sensitivity was severely lacking, in some instances no better than random chance. For the T-stage analysis, with majority of T stage-3 samples not displaying LR (∼86% no-LR and ∼14% LR), it allowed for XGBoost to make the greatest number of correct predictions, evidenced by the high AUROC. On a positive note, XGBoost has learned according to and is consistent with the sample population, evidenced by the consistent higher specificity.

Above considerations notwithstanding, these initial attempts with a supervised learning approach do provide avenues for methodological improvement. These include: (1) larger and more balanced datasets—samples continue to be collected to provide more opportunities for the model to learn; however, emphasis will have to be placed on trying to approach a balanced dataset; (2) distribution-based metrics—statistical features obtained from probability distributions, such as skewness (measure of asymmetry) or kurtosis (measure of “tailedness”) may provide additional insights at the ROI-level that can improve predictive power;^55^ (3) multiple-instance learning—a weakly supervised learning algorithm that deals with data of similar form to this study (i.e., numerous data examples from a set with one associated label)^56^ that may be better suited for this application; and (4) automated ROI selection—a crucial step towards objectivity with this approach and digital histopathology at large, as inconsistent/subjective pathologist ROI selection can negatively affect results. For example, with an ROI selection range of 3 to 14 in this study, patient samples that have far fewer ROIs provide less data when using the majority-vote scheme to inform final patient predictions. Furthermore, manual ROI selection inter-observer variabilities have been reported of upwards of 20% in some studies, not to mention the time-consuming nature of this approach.^57^ Individually or in combination, these avenues will serve as important next steps towards developing a low-cost, alternative PLM-based prognostication tool capable of predicting clinical outcomes in CRC patients and perhaps beyond.

## 4 Conclusion

The value of collagenous stroma within the TNE has been demonstrated across a variety of solid tumors, including CRC. Despite its promise, the lack of a quantitative standardized assessment, among other obstacles, has thwarted its widespread clinical adoption with no clear objective alternatives on the horizon. Findings from this study illustrate the potential value of a polarized light MM imaging + supervised ML platform for predicting 5-year LR status in stage III CRC patients. More specifically, the learning model XGBoost was able to achieve an overall patient-level accuracy of 86% and specificity of 90% when utilizing the most relevant MM features (MM elements $M_{30}$ and $M_{32}$, linear polarization, diattenuation, and linear retardance). The results were further improved to a patient-level accuracy of 96% and specificity of 99% when performing the analysis on the T-stage 3 subset. This demonstrates that useful prognostic information is derivable through polarimetric ML analysis of collagenous stroma, for example in identifying patients with low risk of experiencing LR. These initial promising results need considerable refinement for potential use in clinical settings. Suggested opportunities for improvement include larger and more balanced datasets, distribution-based metrics, automatic ROI segmentation, and multiple-instance learning.

## Data Availability

Code and data underlying the results presented in this paper are not publicly available at this time but may be obtained from the authors upon reasonable request.
